# Primary Prostatic Diffuse Large B‐Cell Lymphoma Mimicking Prostate Cancer With a Normal Prostate‐Specific Antigen Level

**DOI:** 10.1002/iju5.70286

**Published:** 2026-10-26

**Authors:** Kota Amamoto, Yuichiro Nakamura, Hajime Fukushima, Junki Harada, Tsuyoshi Matsuda, Itsuho Ito, Kensuke Mitsunari, Tomohiro Matsuo, Kojiro Ohba, Ryoichi Imamura

**Affiliations:** ^1^ Department of Urology Nagasaki University Graduate School of Biomedical Sciences Nagasaki Japan

**Keywords:** biopsy, large B‐cell lymphoma, prostate, prostate‐specific antigen, prostatic neoplasms

## Abstract

**Introduction:**

Primary prostatic lymphoma is rare and may mimic prostate cancer clinically and radiologically.

**Case Presentation:**

A 66‐year‐old man was referred for evaluation of suspected advanced prostate cancer. Despite a normal prostate‐specific antigen level of 0.573 ng/mL, imaging revealed a prostatic mass with suspected bladder invasion and bilateral hydronephrosis. Prostate needle biopsy showed atypical lymphocytic infiltration but could not distinguish lymphoma from inflammatory changes. Additional transurethral biopsy provided sufficient tissue for histopathological and immunohistochemical evaluation, establishing a diagnosis of primary prostatic diffuse large B‐cell lymphoma.

**Conclusion:**

Primary prostatic lymphoma should be considered when clinical and imaging findings suggest prostate cancer despite a normal prostate‐specific antigen level. Additional tissue sampling should be considered when needle biopsy is inconclusive despite persistent clinical suspicion of lymphoma.

## Keynote Message

- Primary prostatic lymphoma may mimic advanced prostate cancer despite a normal prostate‐specific antigen level.
- Discordance between prostate‐specific antigen and clinical and imaging findings should prompt consideration of lymphoma.
- When needle biopsy is inconclusive despite persistent clinical suspicion, additional tissue sampling, including transurethral biopsy, may facilitate a definitive diagnosis.

**Abbreviations**
CT: computed tomography
DLBCL: diffuse large B‐cell lymphoma
PET/CT: positron emission tomography/computed tomography
PSA: prostate‐specific antigen
R‐CHP: rituximab, cyclophosphamide, doxorubicin, and prednisolone
sIL‐2R: soluble interleukin‐2 receptor

## 1 Introduction

Primary prostatic lymphoma is a rare disease and may be difficult to distinguish from prostate cancer because of similar clinical and imaging findings [1]. We report a case of primary prostatic diffuse large B‐cell lymphoma (DLBCL) with imaging findings suggestive of advanced prostate cancer despite a normal prostate‐specific antigen (PSA) level.

## 2 Case Presentation

A 66‐year‐old man was referred to a local hospital after anemia (hemoglobin, 9.9 g/dL) was detected during a routine health examination. Computed tomography (CT) revealed prostatic enlargement, bladder wall thickening, and bilateral hydronephrosis (Figure 1), prompting referral to our department for suspected advanced prostate cancer. He had experienced difficulty urinating for approximately 5 years and had lost 5 kg over the preceding 3 months. His medical history included hypertension and hyperuricemia. He had no history of lymphoma. Digital rectal examination revealed a stony‐hard prostate with an irregular mass in the right lobe. Magnetic resonance imaging showed a mass predominantly involving the prostatic base, with low signal intensity on T2‐weighted imaging and restricted diffusion (Figure 2). Cystoscopy revealed elevated lesions extending from the posterior to the lateral bladder walls, suggesting bladder invasion (Figure 3).

**FIGURE 1:**
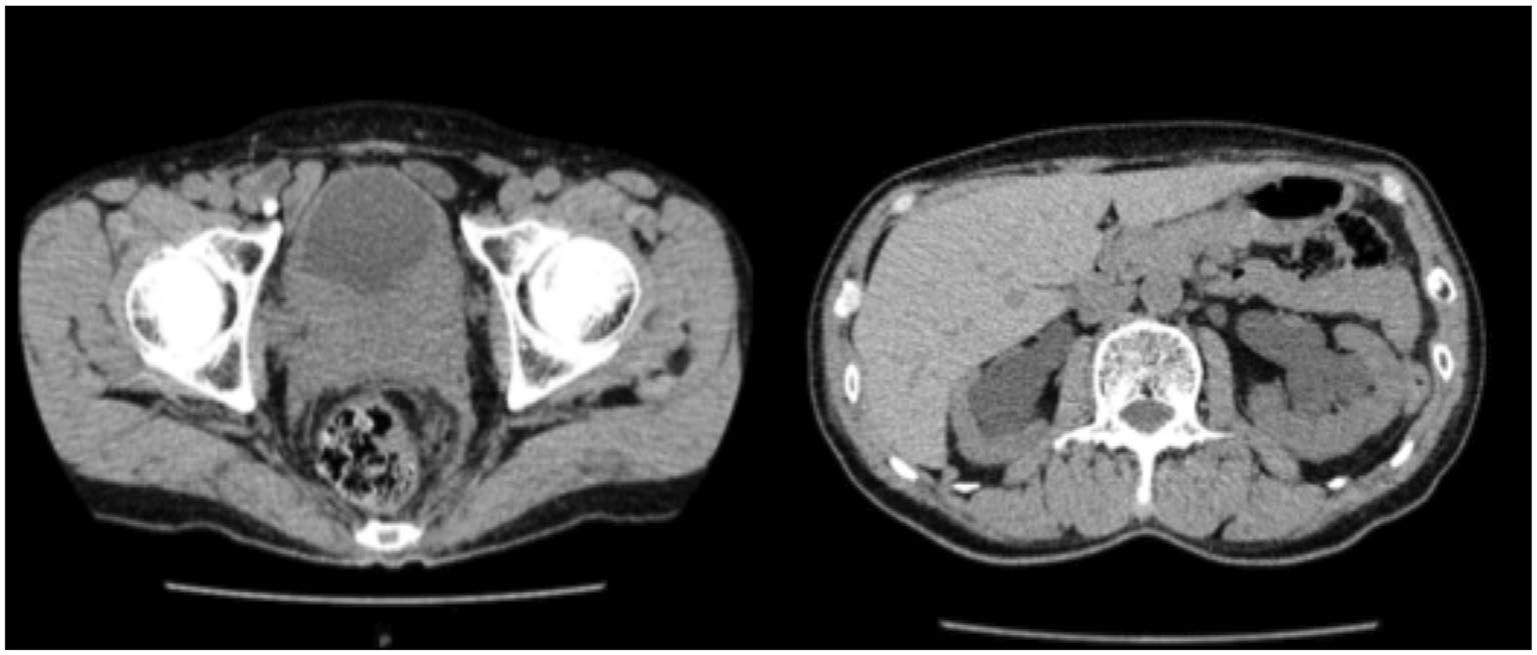
Computed tomography findings at presentation. Computed tomography showing prostatic enlargement, bladder wall thickening, and bilateral hydronephrosis.

**FIGURE 2:**
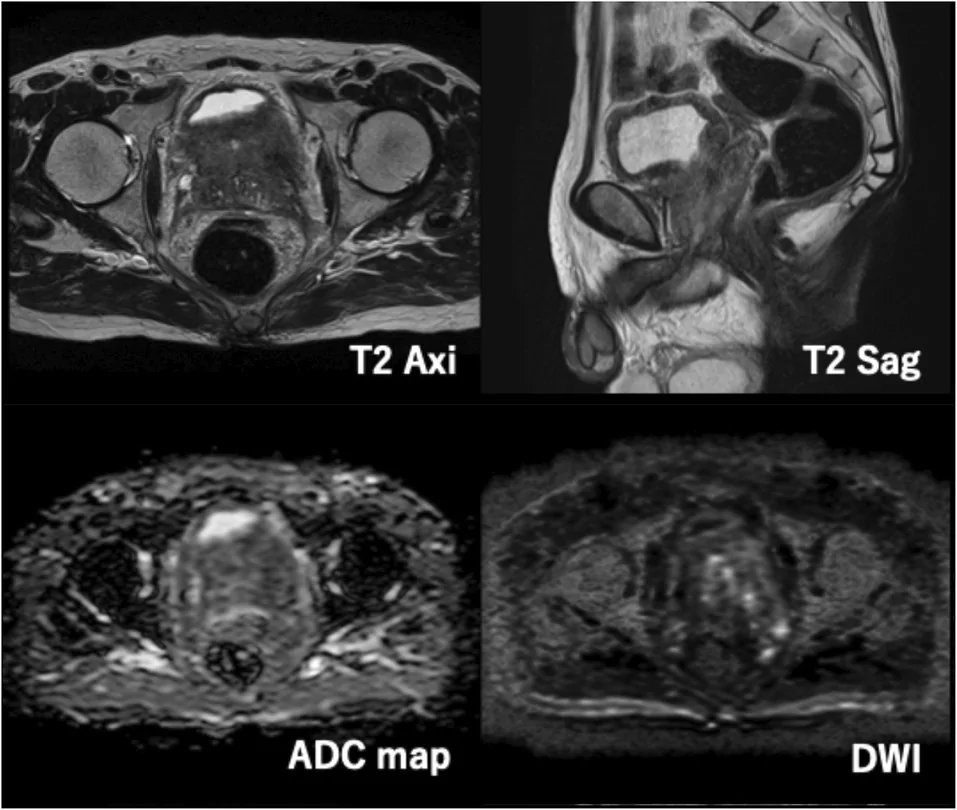
Magnetic resonance imaging findings of the prostate. A mass predominantly involving the prostatic base showing low signal intensity on T2‐weighted imaging and restricted diffusion.

**FIGURE 3:**
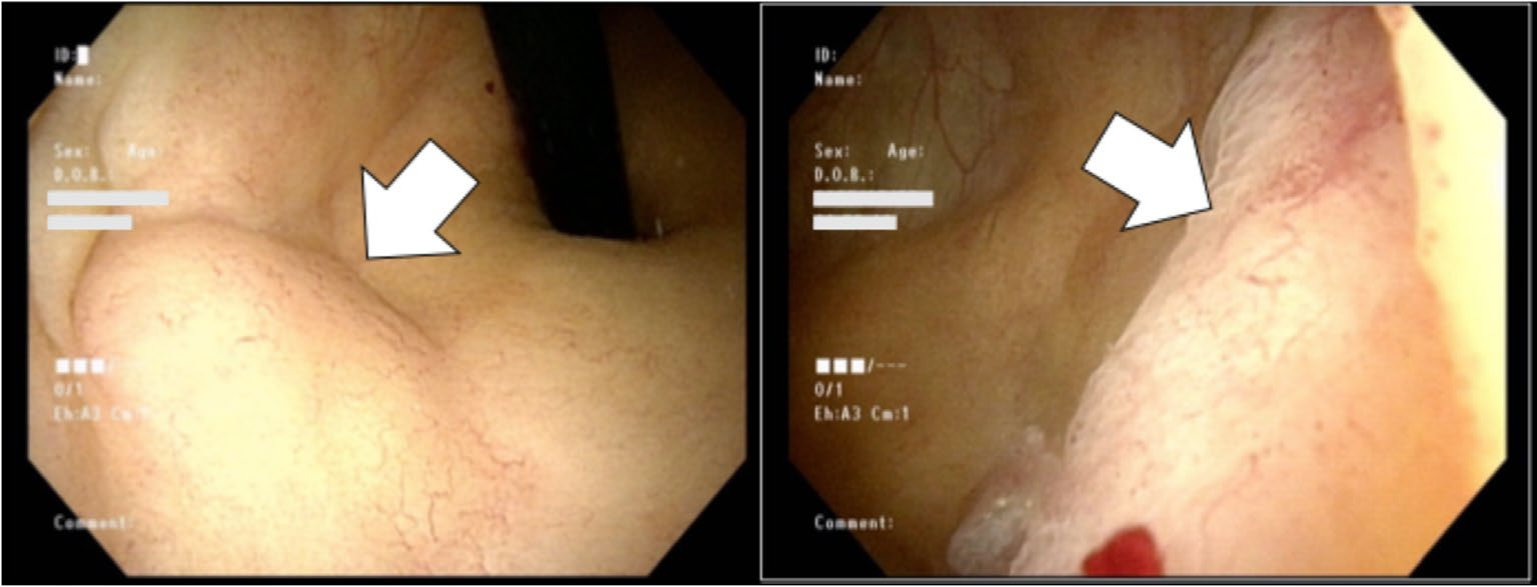
Cystoscopic findings. Elevated lesions extending from the posterior to the lateral bladder walls are observed, suggesting bladder invasion.

At presentation, laboratory tests showed a hemoglobin level of 9.8 g/dL, a white blood cell count of 5900/μL, a platelet count of 27.0 × 10^4^/μL, lactate dehydrogenase of 229 IU/L, C‐reactive protein of 0.03 mg/dL, and creatinine of 1.42 mg/dL. Serum PSA was 0.573 ng/mL, soluble interleukin‐2 receptor (sIL‐2R) was 778 U/mL, neuron‐specific enolase was 8.7 ng/mL, and pro‐gastrin‐releasing peptide was 52.5 pg/mL. The discordance between the PSA level and clinical and imaging findings raised suspicion of malignancy other than conventional prostatic adenocarcinoma, particularly lymphoma. A transrectal 12‐core prostate needle biopsy was performed under conventional ultrasound guidance. Histopathological examination showed interstitial lymphocytic infiltration with some large lymphocytes exhibiting nuclear enlargement and irregular nuclear contours. However, these findings could not be clearly distinguished from inflammatory changes, and an additional transurethral biopsy was performed to obtain sufficient tissue.

Before transurethral biopsy, the hematology department was consulted, and fresh, unfixed tissue was provided to the department for planned flow cytometry, which was not performed. Transurethral samples from the prostate and bladder lesions showed diffuse infiltration and proliferation of large atypical lymphocytes. The tumor cells were positive for CD20, CD10, and BCL6 and negative for MUM1, leading to a diagnosis of the germinal center B‐cell‐like subtype of DLBCL (Figure 4). Positron emission tomography/CT (PET/CT) showed uptake corresponding to the prostatic lesion, without distant lymph node or other organ involvement. The bladder lesion was considered to represent direct extension from the prostate, and primary prostatic DLBCL was diagnosed.

**FIGURE 4:**
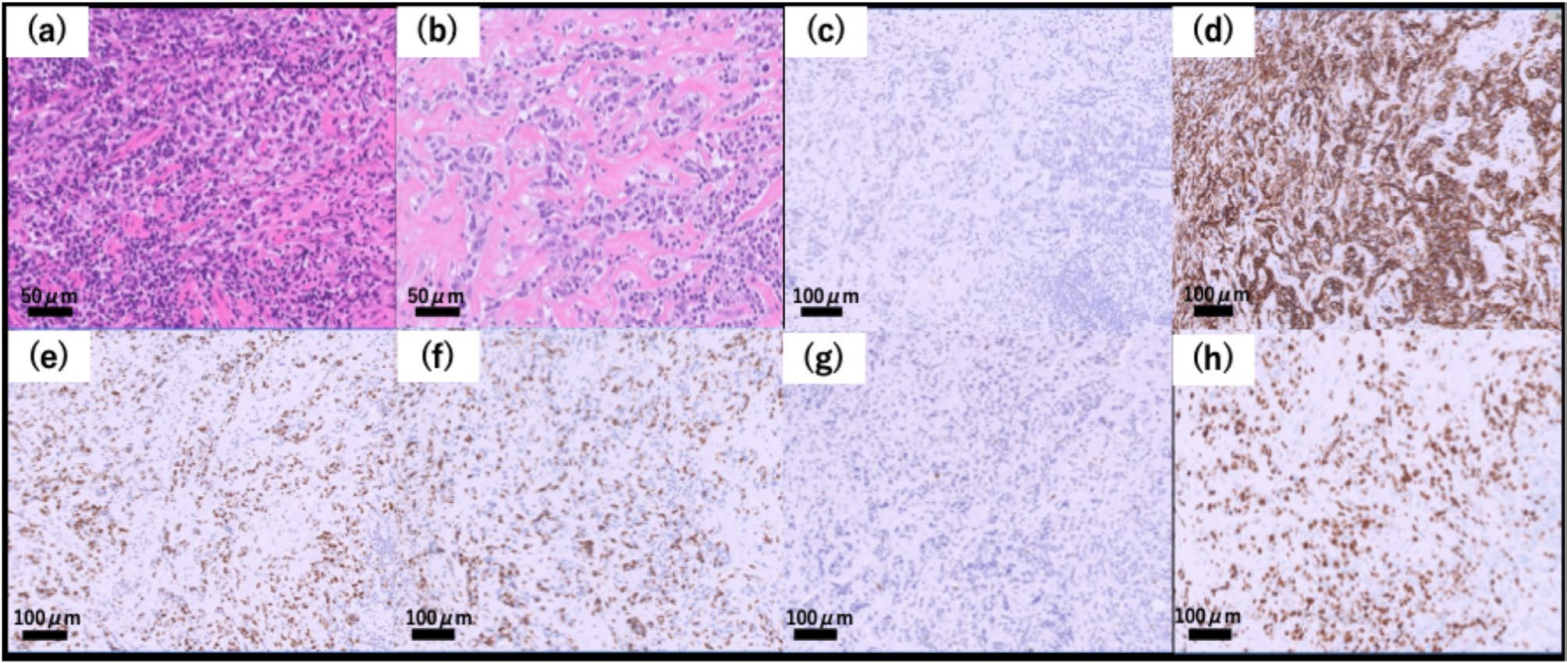
Histopathological findings. (a) Prostate needle biopsy showing interstitial lymphocytic infiltration with some large lymphocytes exhibiting irregular nuclear contours; differentiation from inflammatory changes was difficult (hematoxylin and eosin staining). (b) Transurethral biopsy showing diffuse infiltration and proliferation of large atypical lymphocytes (hematoxylin and eosin staining). Immunohistochemical staining showing positivity for (c) CD10, (d) CD20, and (e) BCL6 and negativity for (f) CD3 and (g) MUM1. (h) Ki‐67 staining showing a high proliferation index.

The patient received eight cycles of systemic therapy including rituximab, cyclophosphamide, doxorubicin, and prednisolone (R‐CHP). Follow‐up PET/CT showed complete resolution of the abnormal uptake in the prostatic lesion. CT performed 6 months after the final rituximab administration showed no evidence of recurrence.

## 3 Discussion

Primary prostatic lymphoma is extremely rare, accounting for approximately 0.09% of prostatic malignancies and 0.1% of non‐Hodgkin lymphomas [2]. Bostwick and Mann proposed three criteria for primary prostatic lymphoma: symptoms attributable to prostatic enlargement; predominant prostatic involvement, with or without adjacent tissue involvement; and no involvement of the liver, spleen, or lymph nodes within 1 month of diagnosis [1, 3]. The present case fulfilled these criteria: urinary symptoms were attributable to prostatic enlargement, the prostate was the predominant site of the disease with direct extension into the bladder, and imaging showed no involvement of the liver, spleen, or lymph nodes within 1 month of diagnosis.

Although clinical and imaging findings may resemble those of prostate cancer, a retrospective analysis of 68 reported cases of primary prostatic DLBCL showed that 89.5% of the 57 patients with available PSA category data had PSA levels < 4 ng/mL [1]. A normal or relatively low PSA level in a patient with a bulky prostatic mass should also prompt consideration of ductal adenocarcinoma and small‐cell neuroendocrine carcinoma. Ductal adenocarcinoma, compared with conventional acinar adenocarcinoma, is associated with lower serum PSA levels, whereas small‐cell carcinoma might present as extensive disease despite low PSA levels [4, 5]. Thus, discordance between PSA and clinical findings should broaden the differential diagnosis and prompt histological evaluation. Serum sIL‐2R is nonspecific and may also be elevated in inflammatory diseases and solid tumors [6]. Its mild elevation in this case did not independently indicate lymphoma but contributed to clinical suspicion alongside the normal PSA level.

Definitive diagnosis requires adequate tissue for histopathological and immunohistochemical evaluation. A systematic review described cases in which prostate needle biopsy yielded benign findings, whereas subsequent transurethral resection specimens established a diagnosis of lymphoma [7]. In the present case, needle biopsy could not distinguish atypical lymphocytic infiltration from inflammatory changes. Additional transurethral biopsy provided sufficient tissue for a definitive diagnosis. Therefore, further tissue sampling, including transurethral biopsy, should be considered when needle biopsy is inconclusive despite persistent clinical suspicion of lymphoma [1, 7].

When lymphoma is suspected, coordination with hematology and pathology departments before biopsy can facilitate appropriate specimen handling, including preservation of fresh, unfixed tissue for flow cytometric immunophenotyping. Flow cytometry may complement morphological and immunohistochemical assessment. In this case, fresh tissue was provided after consultation with hematology, but flow cytometry was not performed; the diagnosis was established by histopathology and immunohistochemistry. This experience highlights the importance of confirming specimen allocation and arrangements for ancillary testing before tissue sampling.

## 4 Conclusion

Primary prostatic lymphoma should be considered when clinical and imaging findings suggest prostate cancer despite a normal PSA level. When prostate needle biopsy is inconclusive despite persistent clinical suspicion of lymphoma, additional tissue sampling, including transurethral biopsy, should be considered.

## Ethics Statement

The authors have nothing to report.

## Consent

Written informed consent was obtained from the patient.

## Conflicts of Interest

The authors declare no conflicts of interest.

## Data Availability

Data sharing not applicable to this article as no datasets were generated or analysed during the current study.
